# A novel multiplex assay for species-level identification of non-tuberculous mycobacteria in culture-positive respiratory specimens based on capillary electrophoresis: development and clinical validation

**DOI:** 10.3389/fmicb.2026.1966137

**Published:** 2026-10-08

**Authors:** Zhen Li, Yaocheng Wang, Li Lai, Yi Huang, Li Li

**Affiliations:** 1 Shengli Clinical Medical College of Fujian Medical University, Fujian Medical University, Fuzhou, China; 2 Tuberculosis Laboratory, International Medical Examination Center, Fuzhou University Affiliated Provincial Hospital, Fuzhou, China; 3 Department of Clinical Laboratory, Fuzhou University Affiliated Provincial Hospital, Fuzhou, China; 4 Department of Health Management, Fuzhou University Affiliated Provincial Hospital, Fuzhou, China

**Keywords:** capillary electrophoresis, mixed-species detection, multiplex assay, NTM, NTM-PD

## Abstract

Nontuberculous mycobacterial pulmonary disease (NTM-PD) is rising globally, driven predominantly by *Mycobacterium avium* complex (MAC), yet treatment is highly species-dependent. We developed a Multiplex assay targeting four clinically important non-tuberculous mycobacteria (NTM) species–*M. avium*, *M. intracellulare*, *M. abscessus*, and *M. fortuitum*–and compared it with metagenomic next-generation sequencing (mNGS), using colony-based multigene Sanger sequencing as the reference method. Among 101 specimens, 63 (62.4%) yielded polymicrobial results, of which mixed-species detection of *M. avium*–*M. intracellulare* accounted for 46 (73.0%). The Multiplex assay and mNGS showed no statistically significant difference in overall accuracy in this cohort (82.2% vs. 81.2%; raw agreement 83.2%, *P* = 1.000). The Multiplex assay achieved 100.0% sensitivity for all four species, with specificity of 87.8% and 87.5% for *M. avium* and *M. intracellulare*, respectively, and >98% for *M. abscessus* and *M. fortuitum*; mNGS showed the converse pattern, with lower MAC sensitivity (88.5%, 92.2%) but higher MAC specificity (93.9%, 95.8%). Requiring only a single PCR reaction without sequencing or bioinformatic analysis, the Multiplex assay showed no statistically significant difference in diagnostic accuracy from mNGS in this culture-positive cohort, without requiring library preparation or sequencing, representing a promising candidate for further evaluation as a routine NTM species-identification tool.

## 1 Introduction

Non-tuberculous mycobacteria pulmonary disease (NTM-PD) represents a growing clinical challenge worldwide with increasing prevalence and significant morbidity. Global epidemiological data indicate that NTM-PD incidence has been rising steadily across multiple regions. In the United States, the annual prevalence of NTM-PD increased from 6.78 to 11.70 per 100,000 population per year between 2008 and 2015 (Winthrop et al., 2020). In Europe, annual prevalence rates vary by country and methodology employed, ranging from 2.3 to 4.5 per 100,000 population per year in the Netherlands (Schildkraut et al., 2021) to 5.3–5.8 per 100,000 population per year in Germany (Corbett et al., 2023). In South Korea, it documented a more than five-fold rise from 6.7 to 39.6 per 100,000 population between 2007 and 2016 (Lee et al., 2019). In China, national and regional surveillance data likewise show a rising NTM isolation rate, from 6.4% of mycobacterial isolates in a 2013 nationwide survey, with a significantly higher proportion of NTM pulmonary disease and rapidly growing mycobacteria in southern and coastal provinces compared with inland and northern regions (Liu et al., 2021). These reflected differences in host susceptibility, environmental mycobacterial ecology and underlying lung disease burden across the region. The true burden may be underestimated due to the absence of mandatory non-tuberculous mycobacteria (NTM) reporting requirements and inconsistent surveillance frameworks across different regions (Daley and Winthrop, 2020).

The diagnosis of NTM-PD remains challenging for several reasons. First, growth characteristics vary substantially among species: slowly growing organisms such as *Mycobacterium avium* complex (MAC) require 8–12 weeks for colony detection on solid media, whereas rapidly growing species typically yield growth within 7 days of subculture (Daley et al., 2020). In addition, NTM-PD presents with clinical features that overlap with other respiratory conditions, and distinguishing colonization from active disease remains difficult (Daley et al., 2020; Dahl et al., 2022). Among the clinically relevant NTM species, *Mycobacterium abscessus*, *Mycobacterium avium*, *Mycobacterium intracellulare*, and *Mycobacterium fortuitum* are frequently implicated in pulmonary infections, with varying geographic distributions and treatment responses (Daley et al., 2020; Dahl et al., 2022). *M. avium* and *M. intracellulare* (collectively referred to as the MAC) account for approximately 50%–91% of NTM-PD cases in North America, with this proportion reaching up to 93% in Japan. Meanwhile, *M. abscessus*/*M. chelonae* account for 2%–18% of isolates and are most frequently identified in the western regions of North America (Hamaguchi et al., 2025; Prevots et al., 2023). These species exhibit distinct drug resistance profiles and clinical outcomes, underscoring the importance of accurate species-level identification (Daley et al., 2020).

Current diagnostic methods for NTM-PD rely heavily on conventional culture techniques, which are time-consuming (1–3 weeks in liquid broth systems and up to 12 weeks on solid media for slowly growing species) and lack intrinsic specificity for species differentiation (Nguyen et al., 2024). Studies have reported sensitivity rates of 63.6%–85.7% for culture-based methods, with significant variability depending on sample quality, processing technique, and the mycobacterial burden of the specimen (Shimoda et al., 2023; Wu et al., 2025). Molecular methods such as Next-Generation Sequencing (NGS) and line probe assays (LPA) have improved detection speed, but present their own limitations, including cost, technical complexity and cross-reactivity. Formal evaluation of the GenoType Mycobacterium CM assay identified cross-reactive hybridization signals in 7 of 14 targeted NTM species (Dohál et al., 2025; Xu et al., 2019).

Capillary electrophoresis (CE) has emerged as a powerful analytical technique with applications in various biomedical fields due to its high resolution, minimal sample requirements, and rapid analysis capabilities (Helena et al., 2022; Kartsova and Makeeva, 2023). Recent advances in CE technology have enhanced its sensitivity and specificity for microbial characterization, making it a promising platform for clinical diagnostics (Gao and Zhong, 2022). Several studies have demonstrated the utility of CE-based approaches for bacterial identification in clinical specimens, with analysis times substantially shorter than those of conventional methods (Horká et al., 2022; Shao et al., 2024). To our knowledge, however, CE-based methodologies have not previously been developed or evaluated for the simultaneous detection and differentiation of multiple clinically relevant NTM species in NTM-PD.

In this study, we present a novel capillary electrophoresis-based approach designed to simultaneously detect four clinically relevant NTM species: *M. abscessus*, *M. avium*, *M. intracellulare* and *M. fortuitum*. We describe the development and optimization of this CE method and evaluate its analytical performance characteristics including sensitivity and specificity. Furthermore, we validate this approach using clinical samples from patients with suspected NTM-PD, comparing its performance against conventional molecular techniques.

## 2 Materials and methods

### 2.1 Primer design

Four oligonucleotide pairs were designed for *M. abscessus*, *M. avium*, *M. intracellulare* and *M. fortuitum*, respectively. Firstly, reference sequences of *16S rRNA*, *HSP65*, *rpoB*, and *dnaA* from seven Mycobacterium species (*M. tuberculosis* ATCC 25618, *M. abscessus* ATCC 19977, *M. avium* ATCC 25291, *M. intracellulare* ATCC 13950, *M. gordonae* ATCC 14470, *M. scrofulaceum* ATCC 19981 and *M. fortuitum* ATCC 6841) were retrieved from NCBI. Then conserved regions were identified through Clustal Omega sequence alignment, followed by primer design using Primer Premier 5 software according to the conserved regions. Forward primer specificity was validated *in silico* via NCBI BLAST. To enable differentiation during electrophoretic analysis, forward primers were 5′-labeled with distinct fluorophores (6-FAM and JOE).

### 2.2 DNA extraction

DNA extraction was performed using a modified Chelex-100 method. Briefly, one milliliter of growth-positive mycobacterial liquid culture was centrifuged at 12,000 rpm for 5 min, and the supernatant was discarded. The pellet was resuspended in 300 μl water containing Chelex resins. To enhance extraction efficiency, 10 μl proteinase K (10 mg/ml) was added to the suspension, considering the high content of lipid in mycobacterium membrane. The mixture was incubated at 56 °C for 20 min followed by heating at 100 °C for 5 min to facilitate DNA release. DNA concentration and purity were subsequently determined using spectrophotometry (NanoDrop One, Thermo Scientific, USA).

### 2.3 Polymerase chain reaction

PCR amplification was performed in 50 μL reaction mixtures containing 1.0 μL dNTP (10 mmol/L) (Invitrogen, USA), 2.0 μL AmpliTaq Gold™ DNA polymerase (5 U/μL) (ABI, USA), 5.0 μL PCR Buffer (without Mg^2+^) (ABI, USA), 8.0 μL MgCl_2_ (25 mmol/L) (ABI, USA), 25.0 μL amplification-grade water (Promega, USA), 0.5 μL of each primer, and 5.0 μL of DNA template. Thermal cycling was conducted on an automated thermal cycler (Verity, ABI, USA) with the following parameters: initial denaturation at 94 °C for 5 min; 35 cycles of denaturation (94°C for 30 s), annealing (55°C for 1 min), and extension (72°C for 1 min); followed by a final extension at 72 °C for 1 min.

### 2.4 Capillary electrophoresis

Capillary electrophoresis was performed using 3130 Genetic Analyzer (ABI, USA). A mixture of LIZ 500 size standard (Promega, USA) and HiDi formamide buffer (Promega, USA) was prepared at 1:130 ratio, and 9.0 μL of this mixture was dispensed into each well of a 96-well plate. PCR products (1.0 μL) were added, followed by denaturation at 99 °C for 3 min. Electrophoretic separation was then conducted according to the manufacturer’s protocol.

### 2.5 Next-generation and Sanger sequencing

To identify potential polymycobacterial infection, growth-positive BACTEC MGIT 960 liquid cultures from patients with suspected NTM-PD were submitted for metagenomic next-generation sequencing (mNGS) at Majorbio (Shanghai, China). Given that residual host nucleic acids from the original clinical specimen may persist in culture fluid, host nucleic acid was depleted by saponin-based selective lysis and DNase I digestion prior to extraction; reads were aligned to the human reference genome (GRCh38) to remove residual host sequences. Then the remaining reads were classified against the NCBI RefSeq microbial genome database. A summary of the mNGS sequencing platform, library preparation, quality-control, and species-assignment parameters used in this study is provided in Supplementary Table 1.

Well-isolated colonies from growth-positive BACTEC MGIT 960 liquid cultures after a solid-culture isolation step were further evaluated using bi-directional sequencing of the *16S rRNA*, *HSP65*, *rpoB*, and *dnaA* gene. Sanger sequencing of targeted DNA was performed by the Sangon Biotech Co., Ltd., (Shanghai, China).

### 2.6 Analytical evaluation

Analytical sensitivity was assessed by determining the limit of detection (LOD) using spiked samples, including *M. abscessus* ATCC 19977, *M. avium* ATCC 25291, *M. intracellulare* ATCC 13950 and *M. fortuitum* ATCC 6841. Twenty replicates at five concentrations approximating the expected LOD were evaluated using purified genomic DNA spiked into a defined matrix. The LOD was defined as the lowest DNA concentration detected in ≥95% of replicates. Analytical specificity was determined by testing 71 microorganisms (Table 1) which represented common respiratory pathogens at following concentrations: bacterial and fungal DNA at 1 × 10^7^ copies/mL, viral nucleic acids at 2 × 10^9^ copies/mL, mycobacterial DNA at 1 × 10^5^ copies/mL, and Chlamydia at 10^6^ elementary bodies/mL. All sensitivity and specificity assays were performed in duplicate.

**TABLE 1:** Microorganisms tested for analytical evaluation.

Microorganisms	Origin	Microorganisms	Origin
*Mycobacterium tuberculosis*	ATCC27294	*Haemophilus influenzae*	Clinical isolates
*Mycobacterium gastri*	ATCC15754	*Haemophilus parainfluenzae*	Clinical isolates
*Mycobacterium terrae*	ATCC15755	*Klebsiella oxytoca*	Clinical isolates
*Mycobacterium xenopi*	ATCC19250	*Klebsiella pneumoniae*	Clinical isolates
*Mycobacterium smegmatis*	ATCC19420	*Legionella pneumophila*	Clinical isolates
*Mycobacterium ulcerans*	ATCC19423	*Micrococcus luteus*	Clinical isolates
*Mycobacterium thermoresistibile*	ATCC19527	*Morganella morganii*	Clinical isolates
*Mycobacterium scrofulaceum*	ATCC19981	*Mycobacterium gordonae*	Clinical isolates
*Mycobacterium simiae*	ATCC25275	*Mycobacterium kansasii*	Clinical isolates
*Escherichia coli*	ATCC25922	*Neisseria meningitidis*	Clinical isolates
*Staphylococcus aureus*	ATCC25923	*Nocardia asteroides*	Clinical isolates
*Pseudomonas aeruginosa*	ATCC27853	*Nocardia brasiliensis*	Clinical isolates
*Mycobacterium haemophilum*	ATCC29548	*Nocardia farcinica*	Clinical isolates
*Mycobacterium malmoense*	ATCC29571	*Proteus mirabilis*	Clinical isolates
*Shigella flexneri*	ATCC29903	*Proteus vulgaris*	Clinical isolates
*Mycobacterium chelonae*	ATCC35752	*Pseudomonas fluorescens*	Clinical isolates
*Mycobacterium szulgai*	ATCC35799	*Pseudomonas putida*	Clinical isolates
*Mycobacterium celatum*	ATCC51131	*Serratia marcescens*	Clinical isolates
*Mycobacterium genavense*	ATCC51234	*Shigella boydii*	Clinical isolates
*Mycobacterium marinum*	ATCC927	*Staphylococcus capitis*	Clinical isolates
*Mycobacterium colombiense*	CIP108962	*Staphylococcus epidermidis*	Clinical isolates
*Mycobacterium paraintracellulare*	JCM30622	*Staphylococcus haemolyticus*	Clinical isolates
*Mycobacterium chimaera*	Clinical isolates	*Staphylococcus saprophyticus*	Clinical isolates
*Acinetobacter baumannii*	Clinical isolates	*Stenotrophomonas maltophilia*	Clinical isolates
*Acinetobacter calcoaceticus*	Clinical isolates	*Streptococcus agalactiae*	Clinical isolates
*Acinetobacter haemolyticus*	Clinical isolates	*Streptococcus mitis*	Clinical isolates
*Acinetobacter junii*	Clinical isolates	*Streptococcus salivarius*	Clinical isolates
*Alcaligenes faecalis*	Clinical isolates	Adenovirus	NIFDC 370057-202001
*Branhamella catarrhalis*	Clinical isolates	Coronavirus	NIFDC 370057-202001
*Candida albicans*	Clinical isolates	Human metapneumovirus	NIFDC 370057-202001
*Citrobacter freundii*	Clinical isolates	Influenza B Virus	NIFDC 370057-202001
*Corynebacterium diphtheriae*	Clinical isolates	Parainfluenza virus	NIFDC 370057-202001
*Enterobacter aerogenes*	Clinical isolates	Respiratory syncytial virus	NIFDC 370057-202001
*Enterobacter cloacae*	Clinical isolates	Rhinovirus	NIFDC 370057-202001
*Enterococcus faecalis*	Clinical isolates	Rubella virus	NIFDC 370057-202001
*Enterococcus faecium*	Clinical isolates		

ATCC, American Type Culture Collection; CIP, Collection of Institut Pasteur, France; JCM, Japan Collection of Microorganisms; DSM, Deutsche Sammlung von Mikroorganismen und Zellkulturen, German; NIFDC, National Institutes for Food and Drug Control, China.

### 2.7 Clinical performance

A cohort study was conducted at Fuzhou University Affiliated Provincial Hospital to compare the diagnostic performance of our Multiplex assay with mNGS for species-level identification of NTM in respiratory specimens. Inclusion criteria: (1) age ≥18 years; (2) clinical suspicion of pulmonary mycobacterial infection; (3) sputum or bronchoalveolar lavage fluid culture-positive by BACTEC MGIT 960; (4) AFB smear-positive and MPB64 antigen-negative on the corresponding positive culture. Exclusion criteria: (1) anti-mycobacterial treatment ≥3 days prior to sampling; (2) colonies after a solid-culture isolation step yield unresolved conflicts by Sanger sequencing; (3) no growth or fewer than 5 isolated colonies obtained on solid subculture. The Institutional Ethics Committee of Fuzhou University Affiliated Provincial Hospital approved the study.

Specimens were cultured using the BACTEC MGIT 960 system. Upon a positive signal, the broth was vortexed and a 1-mL aliquot was centrifuged for AFB smear and MPB64 testing; smear-positive, MPB64-negative specimens were enrolled. For each eligible specimen, the remaining broth was simultaneously aliquoted for mNGS, Multiplex assay, and solid-medium subculture (Lowenstein-Jensen Medium) for reference-standard confirmation. At least five well-isolated colonies per specimen, including any morphologically distinct colonies, were individually sequenced (*16S rRNA*, *HSP65*, *rpoB*, and *dnaA*; bi-directional Sanger sequencing). Species assignment for each colony followed pre-defined identity thresholds for each gene (*16S rRNA*, ≥99.0%; *HSP65*, ≥97.0%; *rpoB*, ≥98.0%; and *dnaA*, ≥97.5%), with the three protein-coding genes prioritized over *16S rRNA* when results conflicted; colonies with unresolved conflicts were excluded. A specimen was classified as monomicrobial if all colonies yielded a single species, or polymycobacterial if two or more species were consistently detected. The confirmed species served as the reference standard for the Multiplex assay and mNGS.

### 2.8 Data analysis

All analyses were performed using R version 4.6.0. Concordance between the Multiplex assay and mNGS was assessed by percent agreement and Cohen’s kappa coefficient. Using the colony-based Sanger sequencing result as the reference standard, the identification accuracy of each method was calculated as the proportion of specimens correctly identified. For polymycobacterial specimens, correct identification required detection of all confirmed species. The paired difference in accuracy between the two methods was compared using McNemar’s test. Sensitivity and specificity of each method were further calculated with their corresponding 95% confidence interval (CI). A two-sided *P* < 0.05 was considered statistically significant.

## 3 Result

### 3.1 CE based Multiplex assay

Specific primers (Table 2) targeted *M. abscessus*, *M. avium*, *M. intracellulare* and *M. fortuitum* were designed based on their conserved region, respectively. Then, the specificity of these forward primers were examined *in silico*. Compared with core nucleotide BLAST database, all four primers showed significant homology restricted to their intended target species or closely related taxa within the same genus, consistent with known phylogenetic relationships among mycobacterial species. For the *M. abscessus*, *M. avium* and *M. intracellulare* primers, a small number of additional matches to phylogenetically unrelated bacterial and non-bacterial taxa were also observed. These taxa were all subsequently included for specificity validation. No off-target matches were observed for the *M. fortuitum* primer. Moreover, the identity of all these matches above is 100%.

**TABLE 2:** Oligonucleotides used for amplification by PCR.

NTM	Gene	Oligonucleotide	Sequence	Fluorophores	Size
*M. abscessus*	*HSP65*	*HSP65*-F	5′-ACCACCGCCACCGTTCTT-3′	5′-6-FAM	241 bp
		*HSP65*-R	5′-CCCTCGTTGCCAACCTTGT-3′	/	
*M. avium*	*rpoB*	*rpoB*-F	5′-GACGGCAATCCCGAGTGG-3′	5′-JOE	235 bp
		*rpoB*-R	5′-CAGGTGGTGCAGCTTCATG-3′	/	
*M. intracellulare*	*16SrRNA*	*16SrRNA*-F	5′-ACCTTTAGGCGCATGTCTT-3′	5′-JOE	330 bp
		*16SrRNA*-R	5′-CTGCTGGCACGTAGTTGG-3′	/	
*M. fortuitum*	*dnaA*	*dnaA*-F	5′-TCCTCTGACGCTGGTTTGTCC-3′	5′-6-FAM	134 bp
		*dnaA*-R	5′-GCGGGCTGCTGAAGTAGGTAG-3′	/	

The result of mixed reference strains comprising *M. abscessus* ATCC 19977, *M. avium* ATCC 25291, *M. intracellulare* ATCC 13950 and *M. fortuitum* ATCC 6841 (DNA concentrations at 1 × 10^3^ copies/mL, respectively) using this Multiplex assay is in line with expectations (Figure 1). After targeted DNA sequencing and alignment with reference sequence, the identity is 99.2% (239/241), 98.7% (232/235), 98.8% (326/330) and 98.5% (132/134) for *M. abscessus*, *M. avium*, *M. intracellulare*, and *M. fortuitum*, respectively (Supplementary Figure 1).

**FIGURE 1:**
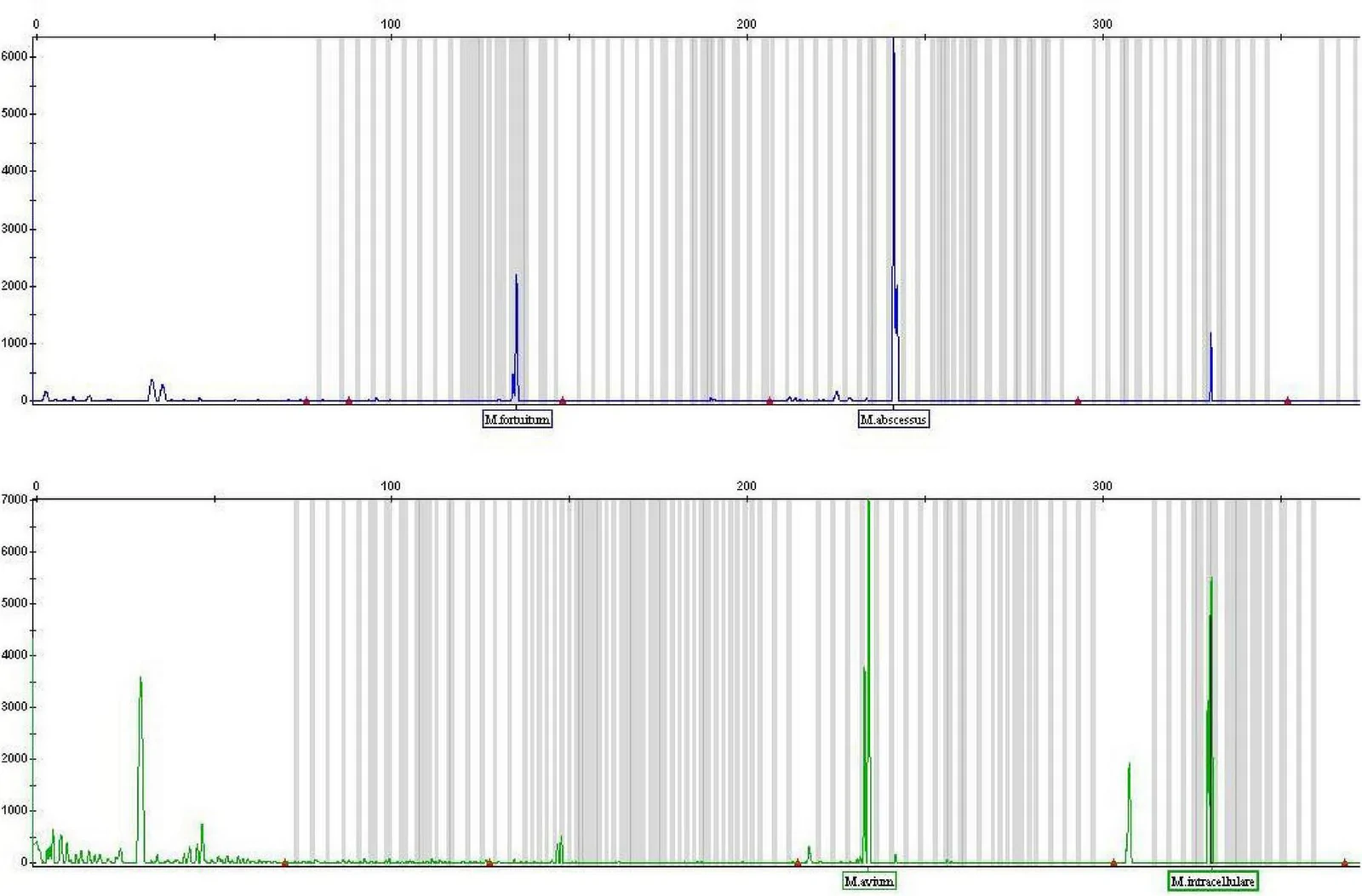
The result of mixed reference strains using Multiplex assay.

### 3.2 Analytical evaluation

All four target NTM species were successfully detected by the Multiplex assay, with LODs of 10–20 copies/mL (Table 3). No amplification was observed from genomic DNA of 70 respiratory pathogens, covering bacteria, fungi, viruses, Chlamydia and other NTM. Consequently, the analytical specificity of the Multiplex assay reached 100% under the given concentrations.

**TABLE 3:** Detection limit of Multiplex assay for four target NTM species.

NTM	Gene	Multiplex assay			
		Limit of detection (copies/ mL)^a^	Number of replicates	Number detected	% detected
*M. abscessus*	*HSP65*	20	20	20	100
*M. avium*	*rpoB*	20	20	19	95
*M. intracellulare*	*16SrRNA*	20	20	20	100
*M. fortuitum*	*dnaA*	10	20	19	95

^a^The limit of detection (LOD) was defined as the lowest DNA concentration that was detected in 95% of 20 replicates.

### 3.3 Clinical performance

A total of 107 respiratory specimens meeting the enrollment criteria (positive BACTEC MGIT 960 culture, positive AFB smear, and negative MPB64 antigen result) were screened between January 2025 and December 2025, of which 6 specimens were excluded because colonies after solid-culture isolation step yield unresolved conflicts by Sanger sequencing. The final analysis therefore included all 101 specimens (Table 4). Of these, 38 were monomicrobial infections and 63 were polymycobacterial, most commonly involving concurrent *M. avium* and *M. intracellulare* (46/63, 73.0%).

**TABLE 4:** Reference standard species composition of enrolled specimens (*N* = 101).

Classification	Species/combination	No. of specimens	% of total
Monomicrobial (*n* = 38)	*M. abscessus*	12	11.9
	*M. avium*	2	2.0
	*M. intracellulare*	17	16.8
	*M. fortuitum*	0	0.0
	Other NTM species^a^	7	6.9
Polymycobacterial (*n* = 63)	*M. avium* + *M. intracellulare*	46	45.5
	*M. abscessus* + *M. fortuitum*	2	2.0
	*M. fortuitum* + *M. intracellulare*	8	7.9
	*M. abscessus* + *M. intracellulare*	1	1.0
	*M. abscessus* + *M. avium* + *M. fortuitum*	1	1.0
	*M. avium* + *M. fortuitum* + *M. intracellulare*	1	1.0
	*M. abscessus* + *M. avium* + *M. intracellulare*	2	2.0
	*M. abscessus* + *M. fortuitum* + *M. intracellulare*	2	2.0
Total		101	100.0

^a^Other NTM species comprised *M. chelonae* (*n* = 3), *M. kansasii* (*n* = 2) and *M. gordonae* (*n* = 2).

Concordance between the Multiplex assay and mNGS is shown in Table 5A. Of the 101 specimens, 74 were correctly identified by both methods and 10 were incorrectly identified by both methods, yielding a percent agreement 83.2%. The Cohen’s kappa coefficient was 0.438 (95% CI 0.194–0.681).

**TABLE 5:** Concordance and diagnostic accuracy of the Multiplex assay and mNGS.

Result	mNGS: correct	mNGS: incorrect	Total	Percent agreement	Cohen’s κ (95% CI)
(A) Concordance between the Multiplex assay and mNGS					
Multiplex assay: correct	74	9	83	83.2%	0.438 (0.194–0.681)
Multiplex assay: incorrect	8	10	18		
Total	82	19	101		
**Method**	**No. correct/total**		**Accuracy, % (95% CI)**		**McNemar’s test**
(B) Diagnostic accuracy against the Sanger sequencing reference standard					
Multiplex assay	83/101		82.2 (73.6–88.4)		χ^2^ = 0.000, *P* = 1.000
mNGS	82/101		81.2 (72.5–87.6)		

The diagnostic accuracy of each method against the reference standard is shown in Table 5B. The Multiplex assay correctly identified 83 of 101 specimens (accuracy 82.2%, 95% CI 73.6%–88.4%), and mNGS correctly identified 82 of 101 specimens (accuracy 81.2%, 95% CI 72.5%–87.6%). The paired difference in accuracy between the two methods was not statistically significant (McNemar’s χ^2^ = 0.000, *P* = 1.000).

Species-specific sensitivity and specificity are presented in Table 6. The Multiplex assay achieved 100.0% sensitivity for all four target species: *M. abscessus* (95% CI 83.9%–100.0%), *M. avium* (95% CI 93.1%–100.0%), *M. intracellulare* (95% CI 95.2%–100.0%) and *M. fortuitum* (95% CI 78.5%–100.0%). The corresponding specificities were 98.8% (95% CI 93.3%–99.8%), 87.8% (95% CI 75.8%–94.3%), 87.5% (95% CI 69.0%–95.7%) and 98.9% (95% CI 93.8%–99.8%), respectively. For mNGS, sensitivity was 95.0% (95% CI 76.4%–99.1%) for *M. abscessus*, 88.5% (95% CI 77.0%–94.6%) for *M. avium*, 92.2% (95% CI 84.0%–96.4%) for *M. intracellulare*, and 100.0% (95% CI 78.5%–100.0%) for *M. fortuitum*, with corresponding specificities of 96.3% (95% CI 89.7%–98.7%), 93.9% (95% CI 83.5%–97.9%), 95.8% (95% CI 79.8%–99.3%) and 98.9% (95% CI 93.8%–99.8%).

**TABLE 6:** Species-specific sensitivity and specificity of the Multiplex assay and mNGS.

Target species (No. of confirmed cases)	Method	Sensitivity, % (95% CI)	Specificity, % (95% CI)
*M. abscessus* (*n* = 20)	Multiplex assay	100.0 (83.9–100.0)	98.8 (93.3–99.8)
	mNGS	95.0 (76.4–99.1)	96.3 (89.7–98.7)
*M. avium* (*n* = 52)	Multiplex assay	100.0 (93.1–100.0)	87.8 (75.8–94.3)
	mNGS	88.5 (77.0–94.6)	93.9 (83.5–97.9)
*M. intracellulare* (*n* = 77)	Multiplex assay	100.0 (95.2–100.0)	87.5 (69.0–95.7)
	mNGS	92.2 (84.0–96.4)	95.8 (79.8–99.3)
*M. fortuitum* (*n* = 14)	Multiplex assay	100.0 (78.5–100.0)	98.9 (93.8–99.8)
	mNGS	100.0 (78.5–100.0)	98.9 (93.8–99.8)

## 4 Discussion

Nontuberculous mycobacterial pulmonary disease has emerged as a growing global health concern, with most surveillance systems reporting a steady rise in both isolation and disease incidence over the past two decades (Dahl et al., 2022; Prevots et al., 2023). This increase is disproportionately driven by MAC (Shah et al., 2016), the two members of which, *M. avium* and *M. intracellulare*, were also the predominant species identified in our cohort. Unlike tuberculosis, for which a single first-line regimen applies broadly, treatment of NTM-PD is highly species-dependent according to the current ATS/ERS/ESCMID/IDSA guideline (Daley et al., 2020). Consequently, an incorrect or incomplete species-level identification does not merely delay diagnosis, it can lead directly to an inappropriate antibiotic regimen. Line probe assays such as GenoType Mycobacterium CM/AS are a molecular alternative to culture-based identification, but published evaluations have documented cross-reactivity among closely related NTM species (Russo et al., 2006). In this study, we developed a Multiplex assay targeting four clinically important NTM species and evaluated it against mNGS, using colony-based multigene Sanger sequencing as the reference method.

To our knowledge, this is the first study to compare a targeted Multiplex assay with mNGS for NTM species identification using a reference method explicitly designed to capture polymycobacterial specimens. Such specimens pose an interpretive challenge for both methods in practice, yet most prior comparative studies have not been designed to resolve them with confidence. Of the 101 enrolled specimens, 63 (62.4%) yielded polymycobacterial results, and *M. avium*–*M. intracellulare* concurrent detection accounted for 46 of these 63 (73.0%). Overall, the Multiplex assay and mNGS showed a raw percent agreement of 83.2% and correctly identified 82.2% and 81.2% of specimens, respectively, a difference that was not statistically significant (McNemar’s χ^2^ = 0.000, *P* = 1.000). However, it is widely acknowledged that mNGS is cumbersome and heavily reliant on specialized bioinformatics analysis. The operational simplicity of Multiplex assay is expected to translate into lower turnaround time, cost and infrastructure requirements, based on typical workflow characteristics for each method (Supplementary Table 2).

In this study, the Multiplex assay achieved 100.0% sensitivity for all four target species, at the cost of comparatively reduced specificity for the two MAC members (87.8% for *M. avium* and 87.5% for *M. intracellulare*), while specificity for *M. abscessus* and *M. fortuitum* remained above 98%. mNGS displayed the converse pattern: lower sensitivity for the MAC members (88.5% and 92.2%, respectively) alongside higher specificity (93.9% and 95.8%). This complementary error structure is mechanistically consistent with how each platform derives its result. The BLAST result had already flagged *M. avium* and *M. intracellulare* as the pair most prone to cross-homology, reflecting their close phylogenetic relationship within MAC. Historically, *M. avium* and *M. intracellulare* were treated as a single taxonomic entity before molecular differentiation became routine. Then, a targeted PCR primer optimized for sensitivity toward one MAC member is inherently more likely to cross-amplify the other. mNGS, in contrast, is not constrained by primer cross-reactivity, but the species differentiation depend on the sequencing depth allocated to each organism present in a specimen (Notarte et al., 2026; Zhao et al., 2023). In a mixed-species specimen, a lower-abundance co-detected MAC species may receive proportionally fewer reads due to competition with the dominant organism for a finite sequencing depth, causing it to fall below the read-count or confidence threshold required for species-level assignment. To investigate whether the reduced clinical specificity for *M. avium* and *M. intracellulare* reflects true primer cross-amplification, we performed two additional analyses. First, wet-laboratory specificity testing of the Multiplex assay against closely related MAC taxa sharing complete forward-primer identity, such as *M. colombiense*, *M. paraintracellulare* and *M. chimaera*, showed no cross-amplification, arguing against a primer-design explanation for the observed false-positive rate. Second, we re-analyzed the raw mNGS sequencing reads already generated from all specimens with a discordant Multiplex-assay/reference-standard result for *M. avium* or *M. intracellulare*, aligning reads under high-stringency parameters to species-specific marker sequences for each organism. For several Multiplex assay positive, reference negative specimens, this re-analysis identified a small number of uniquely mapping reads (approximately 1–5 reads) for the corresponding species, consistent with genuine low-abundance presence that was not captured when only five colonies were selected for Sanger sequencing. We interpret these cases as false negatives of the reference standard rather than false positives of the Multiplex assay. For other discordant specimens, unique *M. intracellulare* reads were also detected, but cross-mapping with extremely close relatives such as *M. chimaera* could not be excluded given the limited resolution of short mNGS reads within highly conserved regions. We classified these cases as ambiguous. Only a minority of discordant specimens showed no corresponding mNGS reads for the non-reference species, consistent with true assay cross-amplification. Full specimen-level results of this arbitration are provided in Supplementary Table 3. On this basis, we consider it likely that the clinical specificity values reported in Table 6 underestimate the assay’s true specificity for *M. avium* and *M. intracellulare*, because a substantial proportion of apparent false positives instead reflect limited sensitivity of the five-colony reference standard for low-abundance co-existing species rather than a shortcoming of the assay itself.

A second finding deserves explicit discussion because it illustrates a common but often overlooked statistical phenomenon. Although raw agreement was 83.2%, Cohen’s kappa was only 0.438, nominally in the “moderate” range. This is not a data quality problem but an example of the kappa paradox: when both raters classify most specimens correctly, the chance-expected agreement (Pe) is pushed toward the observed agreement itself, narrowing the possible range of kappa regardless of the true agreement between methods. In our data, Pe reached 0.701 because both the Multiplex assay (82.2%) and mNGS (81.2%) had high marginal accuracy. With these marginal totals, even substantial true agreement between the two assays could not produce a kappa in the “good” range.

The markedly high rate of polymycobacterial specimens observed here (62.4%) is itself noteworthy and, to our knowledge, higher than rates typically reported in single-organism-focused NTM surveillance series, most of which are not designed to systematically detect mixed-species specimens. We attribute this in part to our reference-standard methodology: we sequenced multiple colonies per specimen rather than a single representative isolate, an approach that would be expected to detect co-existing species that a single-colony or bulk-culture identification strategy would likely miss, misclassifying the specimen as monomicrobial and dominated by the faster- or better-growing organism. We cannot, however, exclude several alternative, non-mutually-exclusive explanations for this high rate: culture-related selection during liquid-to-solid subculture, cross-contamination during colony picking or handling, sampling of only five colonies per specimen, and residual uncertainty around closely related MAC taxa at the reference-standard level. With this caveat, our findings nonetheless raise the possibility that polymycobacterial detection is under-recognized by single-colony identification strategies in routine clinical practice, unless mNGS or multi-colony sequencing was pursued. Whether the specific predominance of concurrent *M. avium*–*M. intracellulare* infection in our cohort reflects local environmental exposure patterns in Fujian Province or a more general biological tendency for MAC members to co-colonize the same airway niche, cannot be resolved and merits further investigation.

This study has several limitations. First, it was conducted at a single center, and the unusually high polymycobacterial rate and species distribution we observed may not generalize to other regions or patient populations; multicenter validation is needed before broader conclusions about assay performance can be drawn. Second, no specimen in our cohort yielded a monomicrobial *M. fortuitum* reference standard. All 14 confirmed *M. fortuitum* cases occurred within polymycobacterial specimens. Although both methods achieved 100% sensitivity for *M. fortuitum* in this context, the assay’s performance against *M. fortuitum* as a sole pathogen remains formally unconfirmed and should be specifically addressed in future validation cohorts. We also note that *M. fortuitum* is a rapidly growing organism with comparatively low intrinsic pathogenicity. Therefore, we regard detection of *M. fortuitum* by our highly sensitive assay in a culture-positive specimen as an early signal prompting confirmation, rather than an independent indication for treatment. Additionally, our assessment of assay specificity against closely related MAC taxa remains limited in scope. Wet-laboratory validation was restricted to a small panel of representative strains (*M. colombiense*, *M. paraintracellulare* and *M. chimaera*), and the in-silico homology analysis was performed primarily on the forward primers rather than on the complete primer set. Given the close phylogenetic relationship among MAC members and the genetic diversity that can exist among clinical isolates of the same species, broader validation against a larger and more genetically diverse panel of MAC isolates would be desirable to more fully define the specificity boundaries of this assay. Finally, our colony-based reference standard, while more rigorous than conventional single-isolate identification, sampled only five colonies per specimen may still under-represent minor co-infecting species due to limited colony sampling and can therefore introduce its own selection bias. Additionally, some species may fall below the analytical recovery threshold of solid culture. This means the true polymycobacterial rate could be even higher than reported here. A more comprehensive identification standard for mixed-species detection is required for further investigation. Additionally, our cohort was restricted to specimens that were MGIT culture-positive, AFB smear-positive, and MPB64-negative; both the Multiplex assay and mNGS were performed on growth-positive culture broth rather than on the primary respiratory specimen. Furthermore, unlike mNGS, the Multiplex assay is an inherently targeted method that can only identify a specimen as one of the four included species; when the assay is negative in a culture-positive specimen, reflex identification by an alternative method (e.g., extended sequencing or mNGS) would still be required. Finally, this single-center study represents an initial proof-of-concept clinical validation; external multicenter validation, interlaboratory reproducibility assessment and prospective evaluation in a less selected diagnostic population are needed before broader clinical implementation can be recommended.

## 5 Conclusion

This study developed and validated a Multiplex assay that showed no statistically significant difference in diagnostic accuracy from mNGS for NTM species identification in this culture-positive cohort, while requiring substantially less operational complexity: a single Multiplex PCR reaction and interpretable result directly from the assay output without the need for library preparation, high-throughput sequencing runs and downstream bioinformatic analysis. Although, external multicenter validation, interlaboratory reproducibility assessment, an expanded specificity panel including closely related MAC taxa, and prospective evaluation in a less selected diagnostic population would be needed before the Multiplex assay could be considered a validated, readily deployable alternative for routine clinical microbiology laboratories, this single-center study provides an initial proof-of-concept clinical validation. As the global burden of NTM-PD continues to rise, a rapid, low-complexity assay for species-level identification of NTM from an already culture-positive specimen represents a useful step toward expediting the microbiologic workup that precedes species-guided treatment decisions.

## Data Availability

The datasets presented in this study can be found in online repositories. The names of the repository/repositories and accession number(s) can be found in the article/Supplementary material.

## References

[B1] Corbett C. Finger P. Heiß-Neumann M. Bohnert J. Eder I. Eisele M. et al. (2023). Development of prevalence and incidence of Non-tuberculous mycobacteria in German laboratories from 2016 to 2020. *Emerg. Microbes Infect.* 12:2276342. 10.1080/22221751.2023.2276342 37883336 PMC10769520

[B2] Dahl V. N. Mølhave M. Fløe A. van Ingen J. Schön T. Lillebaek T. et al. (2022). Global trends of pulmonary infections with Nontuberculous mycobacteria: A systematic review. *Int. J. Infect. Dis.* 125, 120–131. 10.1016/j.ijid.2022.10.013 36244600

[B3] Daley C. L. Winthrop K. L. (2020). Mycobacterium avium complex: Addressing gaps in diagnosis and management. *J. Infect. Dis.* 222, S199–S211. 10.1093/infdis/jiaa354 32814943 PMC7566660

[B4] Daley C. L. Iaccarino J. M. Lange C. Cambau E. Wallace R. Andrejak C. et al. (2020). Treatment of Nontuberculous mycobacterial pulmonary disease: An official ATS/ERS/ESCMID/IDSA clinical practice guideline. *Eur. Respir. J.* 56:2000535. 10.1183/13993003.00535-2020 32636299 PMC8375621

[B5] Dohál M. Wetzstein N. Hromádková M. Mäsiarová S. Rasmussen E. Kunč P. et al. (2025). Diagnostics, resistance and clinical relevance of *Non-tuberculous mycobacteria* unidentified at the species level by line probe assays: A bi-national study. *Ann. Clin. Microbiol. Antimicrob.* 24:14. 10.1186/s12941-025-00781-z 39962513 PMC11834575

[B6] Gao Z. Zhong W. (2022). Recent (2018–2020) development in capillary electrophoresis. *Anal. Bioanal. Chem.* 414, 115–130. 10.1007/s00216-021-03290-y 33754195 PMC7984737

[B7] Hamaguchi Y. Morimoto K. Mitarai S. (2025). Laboratory-based surveillance of Nontuberculous mycobacterial pulmonary disease in Japan. *ERJ Open Res.* 11, 00337–2024. 10.1183/23120541.00337-2024 39963166 PMC11831631

[B8] Helena H. Ivona V. Roman Ř František F. (2022). Current applications of capillary electrophoresis-mass spectrometry for the analysis of biologically important analytes in urine (2017 to mid-2021): A review. *J. Sep. Sci.* 45, 305–324. 10.1002/jssc.202100621 34538010 PMC9292318

[B9] Horká M. Růžička F. Karásek P. Šalplachta J. Siváková A. Roth M. (2022). Classification of clinical *Cutibacterium acnes* isolates at phylotype level by capillary electrophoretic methods in roughened fused silica capillary. *Talanta* 247:123565. 10.1016/j.talanta.2022.123565 35636368

[B10] Kartsova L. Makeeva D. (2023). Biomedical applications of capillary electrophoresis. *J. Anal. Chem.* 78, 1362–1377. 10.1134/s1061934823100118

[B11] Lee H. Myung W. Koh W. Moon S. Jhun B. (2019). Epidemiology of Nontuberculous mycobacterial infection, South Korea, 2007–2016. *Emerg. Infect. Dis.* 25, 569–572. 10.3201/eid2503.181597 30789139 PMC6390769

[B12] Liu C. F. Song Y. M. He W. C. Liu D. He P. Bao J. et al. (2021). Nontuberculous mycobacteria in China: Incidence and antimicrobial resistance spectrum from a nationwide survey. *Infect. Dis. Poverty* 10:59. 10.1186/s40249-021-00844-1 33926548 PMC8082609

[B13] Nguyen M. H. Haas M. K. Kasperbauer S. H. Calado Nogueira de Moura V. Eddy J. J. Mitchell J. D. et al. (2024). Nontuberculous mycobacterial pulmonary disease: Patients, principles, and prospects. *Clin. Infect. Dis.* 79, e27–e47. 10.1093/cid/ciae421 39405483

[B14] Notarte K. I. Allen K. Armstrong D. Parrish N. (2026). Evaluation of the deeplex Myc-TB-targeted next-generation sequencing assay for mycobacterial identification and drug resistance detection. *J. Clin. Microbiol.* 64:e0008226. 10.1128/jcm.00082-26 42214384 PMC13343837

[B15] Prevots D. R. Marshall J. E. Wagner D. Morimoto K. (2023). Global epidemiology of Nontuberculous mycobacterial pulmonary disease: A review. *Clin. Chest Med.* 44, 675–721. 10.1016/j.ccm.2023.08.012 37890910 PMC10625169

[B16] Russo C. Tortoli E. Menichella D. (2006). Evaluation of the new GenoType Mycobacterium assay for identification of mycobacterial species. *J. Clin. Microbiol.* 44, 334–339. 10.1128/JCM.44.2.334-339.2006 16455880 PMC1392669

[B17] Schildkraut J. A. Zweijpfenning S. H. M. Nap M. He K. Dacheva E. Overbeek J. et al. (2021). The epidemiology of Nontuberculous mycobacterial pulmonary disease in the Netherlands. *ERJ Open Res*. 7:00207-2021. 10.1183/23120541.00207-2021 34262970 PMC8273392

[B18] Shah N. M. Davidson J. A. Anderson L. F. Lalor M. Kim J. Thomas H. et al. (2016). Pulmonary Mycobacterium avium-intracellulare is the main driver of the rise in *Non-tuberculous mycobacteria* incidence in England, Wales and Northern Ireland, 2007–2012. *BMC Infect. Dis.* 16:195. 10.1186/s12879-016-1521-3 27154015 PMC4858927

[B19] Shao Y. C. Wen Y. L. Zhao X. Y. Qu F. (2024). Annual review of capillary electrophoresis technology in 2023. *Chinese J. Chromatogr.* 42, 401–409. 10.3724/sp.j.1123.2024.02007 38736383 PMC11089452

[B20] Shimoda M. Morimoto K. Yoshiyama T. Tanaka Y. Furuuchi K. Fujiwara K. et al. (2023). Usefulness of gastric aspirates for diagnosing *Nontuberculous mycobacteriosis*. *Sci. Rep.* 13:7858. 10.1038/s41598-023-34948-5 37188839 PMC10185579

[B21] Winthrop K. L. Marras T. K. Adjemian J. Zhang H. Wang P. Zhang Q. (2020). Incidence and prevalence of Nontuberculous mycobacterial lung disease in a large U.S. managed care health plan, 2008–2015. *Ann. Am. Thorac. Soc.* 17, 178–185. 10.1513/AnnalsATS.201804-236OC 31830805 PMC6993793

[B22] Wu Q. Zhu Y. Zhang Y. Liu Z. Zhang M. Chen J. et al. (2025). Evaluation and comparison of laboratory methods in diagnosing *Mycobacterium tuberculosis* and Nontuberculous mycobacteria in 3012 sputum samples. *Clin. Respir. J.* 19:e70071. 10.1111/crj.70071 40087831 PMC11908973

[B23] Xu Y. Liang B. Du C. Tian X. Cai X. Hou Y. et al. (2019). Rapid identification of clinically relevant mycobacterium species by multicolor melting curve analysis. *J. Clin. Microbiol.* 57:e01096-18. 10.1128/JCM.01096-18 30333128 PMC6322455

[B24] Zhao Z. Chen X. Wang Y. Feng J. (2023). Comparison of quality/quantity mNGS and usual mNGS for pathogen detection in suspected pulmonary infections. *Front. Cell. Infect. Microbiol.* 13:1184245. 10.3389/fcimb.2023.1184245 37588054 PMC10425550

