# Supplementary material for: A novel multiplex assay for species-level identification of non-tuberculous mycobacteria in culture-positive respiratory specimens based on capillary electrophoresis: development and clinical validation
Source: Front Microbiol. 2026 Oct 8;17:1966137. doi: 10.3389/fmicb.2026.1966137 (PMC13687058; doi:10.3389/fmicb.2026.1966137)
Supplement: Supplementary file 1 [file Data_Sheet_1.pdf]

TABLE S1 mNGS Methodological Parameters

Parameter	Specification
Sequencing platform	Illumina NovaSeq 6000 (PE150)
Library preparation kit	Illumina DNA Prep Kit
Host depletion method	saponin-based selective lysis and DNase I digestion
Minimum raw reads/sample	≥10 million
Quality filtering	Cutadapt (adapter removal); Q20 filter; remove <35 bp reads; remove duplicates
Host read removal	bwa- mem2 alignment to GRCh38
QC acceptance	>75% reads with Q30
MTBC positive threshold	SMRN ≥1
NTM/bacteria positive threshold	SMRN ≥3 (or RPM ≥1)
Virus positive threshold	≥3 non- overlapping reads
Genome coverage requirement	≥3 non- overlapping regions
MAC species discrimination	SMRN with ≥97% identity, ≥80% coverage; ambiguous reads reported at complex level
Contamination control	NTC per batch; report only if SMRN exceeds NTC

TABLE S2 Operational comparison between the Multiplex assay and mNGS

Parameter	Multiplex assay (Capillary electrophoresis)	mNGS
Hands-on time per specimen	~15–20 min per specimen for DNA extraction and PCR setup; approximately 45–60 min total hands-on time per batch of 16–24 specimens	Approximately 4–6 h per batch for nucleic acid extraction, host depletion, library preparation, and quantification, largely independent of specimen number within a batch
Total turnaround time (from growth-positive culture to result)	Approximately 4–6 h from growth-positive culture to result (extraction, PCR, capillary electrophoresis, and fragment-size analysis)	Approximately 24–48 h from growth-positive culture to report (library preparation, sequencing run, and bioinformatic analysis)
Batch size per run	Typically 16–24 specimens per run, depending on the capillary genetic analyzer used	Typically 8–24 barcoded specimens multiplexed per sequencing run, depending on the sequencing platform
Equipment required	Thermal cycler and capillary genetic analyzer; no sequencing or bioinformatics infrastructure needed	Next-generation sequencing platform, host-depletion/library-preparation reagents and a bioinformatics pipeline for taxonomic classification
Analysis required after run completion	Fragment-size calling against pre-defined size standards; no sequence alignment or bioinformatics pipeline	Read quality filtering, host-sequence removal and taxonomic classification against a reference microbial genome database
Approximate reagent cost per sample	Low; approximately US$5–15 per sample for PCR reagents and capillary electrophoresis consumables	Substantially higher; approximately US$150–300 per sample for library preparation and sequencing reagents

TABLE S3 Discordance Resolution for *M. avium* / *M. intracellulare* Using mNGS Re-analysis

Specimen ID	Multiplex Assay Result	Sanger Reference Result	mNGS Re-analysis (Unique reads mapping to *M. avium* / *M. intracellulare*)	Adjudication	Interpretation
S-007	*M. avium* (+)	Negative (only *M. intracellulare* found)	3 reads uniquely mapping to *M. avium*; 0 reads for *M. intracellulare*	True positive (Multiplex), false negative (Sanger)	Low-abundance *M. avium* missed by colony picking; not primer cross-reactivity
S-019	*M. intracellulare* (+)	Negative (only *M. avium* found)	0 reads for *M. intracellulare*; 12 reads for *M. avium*	False positive (Multiplex)	No evidence of *M. intracellulare* in mNGS;<br>likely cross-amplification with *M. avium*
S-033	*M. avium* (+)	Negative (only *M. intracellulare* found)	2 reads mapping to *M. avium* and 1 read mapping to *M. chimaera*	Indeterminate	Low reads for both; short-read mNGS cannot confidently distinguish *M. avium* from *M. chimaera*; classified as ambiguous
S-041	*M. intracellulare* (+)	Negative (only *M. avium* found)	5 reads for *M. intracellulare*	True positive (Multiplex), false negative (Sanger)	*M. intracellulare* missed by colony picking; not primer cross-reactivity

Continued

S-052	*M. avium* (+)	Negative (Sanger found only *M. abscessus*)	3 reads for *M. avium*; 20 reads for *M. abscessus*	True positive (Multiplex), false negative (Sanger)	Low-abundance *M. avium* missed by dominant mycobacteria
S-057	*M. avium* (+)	Negative (only *M. intracellulare* found)	3 reads mapping to *M. avium* and 1 read mapping to *M. chimaera*	Indeterminate	Low reads for both; short-read mNGS cannot confidently distinguish *M. avium* from *M. chimaera*; classified as ambiguous
S-072	*M. avium* (+)	Negative (only *M. intracellulare* found)	4 reads uniquely mapping to *M. avium*; 0 reads for *M. intracellulare*	True positive (Multiplex), false negative (Sanger)	Low-abundance *M. avium* missed by colony picking; not primer cross-reactivity
S-087	*M. intracellulare* (+)	Negative (only *M. avium* found)	0 reads for *M. intracellulare*; 8 reads for *M. avium*	False positive (Multiplex)	No evidence of *M. intracellulare* in mNGS;<br>likely cross-amplification with *M. avium*
S-094	*M. avium* (+)	Negative (only *M. intracellulare* found)	3 reads uniquely mapping to *M. avium*; 0 reads for *M. intracellulare*	True positive (Multiplex), false negative (Sanger)	Low-abundance *M. avium* missed by colony picking; not primer cross-reactivity
