# Supplementary material for: A novel multiplex assay for species-level identification of non-tuberculous mycobacteria in culture-positive respiratory specimens based on capillary electrophoresis: development and clinical validation
Source: Front Microbiol. 2026 Oct 8;17:1966137. doi: 10.3389/fmicb.2026.1966137 (PMC13687058; doi:10.3389/fmicb.2026.1966137)
Supplement: Supplementary file 2 [file Image_1.pdf]

A

```
CLUSTAL O(1.2.4) multiple sequence alignment


HSP65           ACCACCGCCACCGTTCTTGCCCAGGCCCTGGTCAAGGAAGGTCTGCGTAACGTCGCCGCC    60
AY458075.1      ACCACCGCCACCGTTCTTGCCCAGGCCCTGGTCAAGGAAGGTCTGCGTAACGTCGCCGCC    60
                ************************************************************

HSP65           GGCGCCAACCCGCTCGGCCTGAAGCGCGGCATCGAGAAGGGCGTCGAGAAGGTCACGGAG    120
AY458075.1      GGCGCCAACCCGCTCGGCCTGAAGCGCGGCATCGAGAAGGCCGTCGAGAAGGTCACCGAG    120
                **************************************** *************** ***

HSP65           ACGCTGCTGAAGAGCGCCAAGGAGGTCGAGACCAAGGAGCAGATCGCGGCCACGGCCGGT    180
AY458075.1      ACGCTGCTGAAGAGCGCCAAGGAGGTCGAGACCAAGGAGCAGATCGCGGCCACGGCCGGT    180
                ************************************************************

HSP65           ATCTCCGCGGGCGACCAGTCCATCGGATCGCCGAGGCCATGGACAAGGTTGGCAACGAGG    240
AY458075.1      ATCTCCGCGGGCGACCAGTCCATCGGATCGCCGAGGCCATGGACAAGGTTGGCAACGAGG    240
                ************************************************************

HSP65           G       241
AY458075.1      G       241
                *
```

B

```
CLUSTAL O(1.2.4) multiple sequence alignment


rpoB            GACGGCAATCCCGAGTGGGCGGTCAACCTGCCCGAGGAGCTGCGGCACGCACAGCCGAAC    60
EF521907.1      GACGGCAATCCCGAGTGGGCGGTCAACCTGCCCGAGGAGCTGCGGCACGCACAGCCGAAC    60
                ************************************************************

rpoB            CAGATCGTGTCGACACCGGTGTTCGACGGCGCCAAGGAAGAGGACCTGGCCGGCATCCTG    120
EF521907.1      CAGATCGTGTCGACACCGGTGTTCGACGGCGCCAAGGAAGAGGAGCTGGCCGGCATGCTG    120
                ******************************************** *********** ***

rpoB            TCGTGCACGCTCCCCAACCGCGACGGCGAGGTCATGGTGGACGGCGACGGCAAGGGAGCC    180
EF521907.1      TCGTGCACGCTGCCCAACCGCGACGGCGAGGTCATGGTGGACGGCGACGGCAAGGGAGCC    180
                *********** ************************************************

rpoB            GTTCCCGTACCCGGTGACCGTCGGCTACATGTACATCATGAAGCTGCACCACCTG 235
EF521907.1      GTTCCCGTACCCGGTGACCGTCGGCTACATGTACATCATGAAGCTGCACCACCTG 235
                *******************************************************
```

C

```
CLUSTAL O(1.2.4) multiple sequence alignment


16SrRNA         ACCTTTAGGCGCATGTCTTTAGGTGGAAAGCTTTTGCGGTGTGGGATGGGCCCGCGGCCT   60
NR_029003.1     ACCTTTAGGCGCATGTCTTTAGGTGGAAAGCTTTTGCGGTGTGGGATGGGCCCGCGGCCT   60
                ************************************************************

16SrRNA         ATCAGCTTGTTGGTGGGGTGATGGCCTACCAAGGCGACGACCGGTAGCCGGCCAGAGAGG   120
NR_029003.1     ATCAGCTTGTTGGTGGGGTGATGGCCTACCAAGGCGACGACGGGTAGCCGGCCTGAGAGG   120
                ***************************************** *********** ******

16SrRNA         GTGTCGGGCCAGACTGGGACTGAGATACGGCCCAGACTCCTACGGGAGGCAGCAGGAATA   180
NR_029003.1     GTGTCCGGCCACACTGGGACTGAGATACGGCCCAGACTCCTACGGGAGGCAGCAGGAATA   180
                ***** ***** ************************************************

16SrRNA         TTGCACAATGGGCGCAAGCCTGATGCAGCGACGCCGCGTGGGGGATGACGGCCTTCGGGT   240
NR_029003.1     TTGCACAATGGGCGCAAGCCTGATGCAGCGACGCCGCGTGGGGGATGACGGCCTTCGGGT   240
                ************************************************************

16SrRNA         TGTAAACCTCTTTCACCATCGACGAAGGTTCGGGTTTTCTCGGATTGACGGTAGGTGGAG   300
NR_029003.1     TGTAAACCTCTTTCACCATCGACGAAGGTTCGGGTTTTCTCGGATTGACGGTAGGTGGAG   300
                ************************************************************

16SrRNA         AAGAAGCACCGGCCAACTACGTGCCAGCAG 330
NR_029003.1     AAGAAGCACCGGCCAACTACGTGCCAGCAG 330
                ******************************
```

D

```
CLUSTAL O(1.2.4) multiple sequence alignment


dnaA            TCCTCTGACGCTGGTTTGTCCGACGACGAACGTCCAGACCCGAGCGCTGCCACGCCGGGC    60
AB087689.1      TCCTCTGACGCTGGTTTGTCCGACGACGAACGTCCAGACGCGAGCGCTGCCACGCCGGCC    60
                *************************************** ****************** *

dnaA            GCCGACCTCGACGAGATCGACGACGACCGCGCCGAGCGCCGAGGAATCATGGCCTACCTA    120
AB087689.1      GCCGACCTCGACGAGATCGACGACGACCGCGCCGAGCGCCGAGGAATCATGGCCTACCTA    120
                ************************************************************

dnaA            CTTCAGCAGCCCGC  134
AB087689.1      CTTCAGCAGCCCGC  134
                **************
```

Fig. S1. The alignment with reference sequence. (A) *M.abscessus (HSP65)*. (B) *M. avium (rpoB)*. (C) *M. intracellulare (16SrRNA)*. (D) *M. fortuitum (dnaA)*.
